# Challenges in the Treatment of Glioblastoma by Chimeric Antigen Receptor T-Cell Immunotherapy and Possible Solutions

**DOI:** 10.3389/fimmu.2022.927132

**Published:** 2022-07-07

**Authors:** Peng Zhang, Yang Zhang, Nan Ji

**Affiliations:** ^1^Department of Neurosurgery, Beijing Tiantan Hospital, Capital Medical University, Beijing, China; ^2^China National Clinical Research Center for Neurological Diseases, Beijing, China; ^3^Beijing Advanced Innovation Center for Big Data-Based Precision Medicine, Beihang University, Beijing, China

**Keywords:** glioblastoma, chimeric antigen receptor, CAR-T, adoptive immunotherapy, cellular immunotherapy

## Abstract

Glioblastoma (GBM), one of the most lethal brain cancers in adults, accounts for 48.6% of all malignant primary CNS tumors diagnosed each year. The 5-year survival rate of GBM patients remains less than 10% even after they receive the standard-of-care treatment, including maximal safe resection, adjuvant radiation, and chemotherapy with temozolomide. Therefore, new therapeutic modalities are urgently needed for this deadly cancer. The last decade has witnessed great advances in chimeric antigen receptor T (CAR-T) cell immunotherapy for the treatment of hematological malignancies. Up to now, the US FDA has approved six CAR-T cell products in treating hematopoietic cancers including B-cell acute lymphoblastic leukemia, lymphoma, and multiple myeloma. Meanwhile, the number of clinical trials on CAR-T cell has increased significantly, with more than 80% from China and the United States. With its achievements in liquid cancers, the clinical efficacy of CAR-T cell therapy has also been explored in a variety of solid malignancies that include GBMs. However, attempts to expand CAR-T cell immunotherapy in GBMs have not yet presented promising results in hematopoietic malignancies. Like other solid tumors, CAR-T cell therapies against GBM still face several challenges, such as tumor heterogeneity, tumor immunosuppressive microenvironment, and CAR-T cell persistence. Hence, developing strategies to overcome these challenges will be necessary to accelerate the transition of CAR-T cell immunotherapy against GBMs from bench to bedside.

## Introduction

Chimeric antigen receptor T (CAR-T) cell is a genetically engineered T lymphocyte that expresses CAR molecules to target surface antigens on tumor cells and other cells. The CAR construct is composed of an extracellular antigen-binding domain (the majority is the variable domain of an antibody targeting the antigen), an intracellular signal transduction domain, and a transmembrane domain that links the extracellular and intracellular domains (1). With the innovations in the design of the CAR structure, CARs have evolved from first to fifth generation. The intracellular domain of first-generation CAR only contains the CD3ζ chain and original signal transmitters from native T-cell receptor signaling that will limit T-cell activation and thus reduce its antitumor efficacy (2–4). Therefore, the second- and third-generation CARs have been developed to enhance T-cell activation by incorporating one and two intracellular signaling domains of co-stimulatory molecules, respectively, into its cytoplasmic tail. These co-stimulatory molecules include CD28 and ICOS from the B7 family, and OX-40 and 4-1BB from the tumor necrosis factor receptor (TNFR) family. Among them, the intracellular domains of CD28 and 4-1BB are commonly used in the CAR construct. Given that they belong to different families with distinct downstream signaling (5), the CD28 or 4-1BB incorporated second-generation CAR, termed CD28-CAR or 4-1BB-CAR, showed distinct signaling kinetics in T-cell activation with the CD28-CAR displaying a faster and enhanced change in protein phosphorylation than the 4-1BB-CAR. Consequently, the CD28-CAR-T cells exhibit a robust but short-lived effector T-cell phenotype, whereas the 4-1BB-CAR-T cells preferentially express memory T-cell genes, leading to more longevity of the CAR-T cells as well as sustained antitumor activities (6, 7). Although the third-generation CAR contains two different co-stimulatory domains (e.g., from CD28 and 4-1BB), it did not show significant advantages over the second-generation CAR in antitumor response (8–10). The fourth-generation CAR-T cell therapy refers to the second-generation CAR-T cells armed with immune stimulatory cytokines (e.g., IL-12, IL-15, and IL-18) that are released while they engage targeted tumor cells, leading to the improvement of CAR-T cell expansion/persistence as well as the promotion of the antigen spreading by recruiting endogenous T cells or NK cells (11–16). Moreover, the encouraging preclinical results of fourth-generation CAR has renewed interest in the concept of “targeted cellular micropharmacies” (17, 18), which utilize immune cells as a tumor-targeted living carrier to deliver therapeutic agents, including antibodies, enzymes, immunostimulatory molecules, as well as nanoparticles loaded with anti-cancer drugs (17, 18). The structure of the fifth-generation CAR is also based on the second-generation CAR, but with the addition of truncated cytoplasmic domains of cytokine receptors and a STAT3-binding motif (19) that permits cytokine engagement signaling (signal 3), resulting in the optimization of T-cell activation and thus superior *in vivo* persistence and antitumor effects in preclinical models as compared with the second-generation CAR (19).

**Figure 1:**
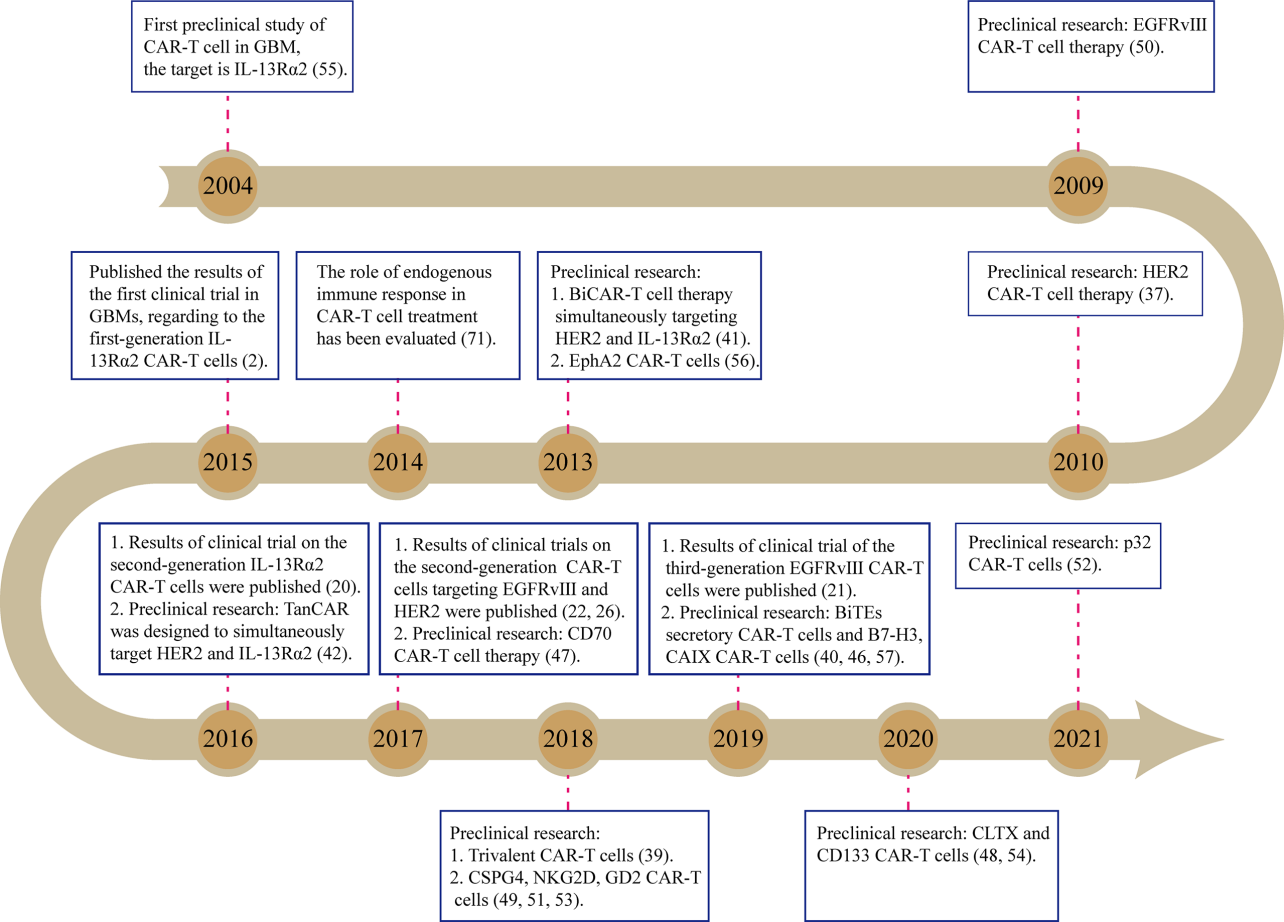
The main course of developing CAR-T cell therapies on GBMs.

Until now, there have been dozens of preclinical studies as well as several clinical trials on the glioblastoma (GBM) treatment by CAR-T cells, attempting to target various tumor antigens (**Figure 1**). Among them, EGFR-vIII, HER2, and IL13Rα2 are the three most common targets and have been tested in early phase trials (**Table 1**, **Supplementary Table 1**). IL13Rα2-targeted CAR-T cells are reportedly the first product to enter the trial stage (2). In this trial, three patients with recurrent GBMs were treated by intracerebroventricular injection of first-generation CAR-T cells that were engineered to express IL-13 zetakine, a mutant ligand to IL13Rα2 (NCT00730613) (2). Although a peritumoral inflammatory response was observed by MRI in all the patients, tumor regression was quite transient, thus requiring continuous CAR-T cell delivery (2). The second-generation IL13Rα2-targeted CAR-T cells (4-1BB-CAR-T) were also delivered *via* multiple intracranial injections to treat one patient with recurrent GBMs (NCT02208362) (20). Two intracranial delivery routes—injecting directly into the resected tumor cavity followed by infusing into the ventricular system—were applied in this study to control tumor local recurrence and tumor dissemination, respectively (20). As a result, regression of all intracranial and spinal tumors was observed after multiple injections, along with the corresponding immune response detected in the cerebrospinal fluid. The complete response lasts for 7.5 months after the initiation of CAR-T cell therapy. Meanwhile, no ≥ CTCAE (Common Terminology Criteria for Adverse Events) grade 3 adverse events, related to the CAR-T cell therapy, were observed during the treatment (20). However, the HER2 (NCT01109095)- and EGFR-vIII (NCT02209376 and NCT01454596)-targeted CAR-T cell therapies were administered through intravenous infusions (21, 22) in their early-stage clinical trials. Both trials show that intravenously infused CAR-T cells are capable of migrating into brain tumors through the blood–brain barrier (BBB), exerting antigen-specific tumor killing, reducing tumor volume, and thereby extending survival time in a fraction of GBM patients (21, 22).

**Table 1:** Published clinical trials of CAR-T in treating adult GBMs.

ClinicalTrials.gov identifier; Start-completed/terminated year;status	Target antigen	Number of patients enrolled	CAR-T design	Dosage of CAR-T cells and route of administration	Adverse effects (≥ CTCAE grade 3) that possibly related to CAR-T therapies	Clinical outcomes
NCT00730613; 2002-2011; Completed	IL-13Ra2 (2)	3	First-generation CAR on CD8+ T cells	10^7^–10^8^ for 11–12 intra-cavitary inj.; 10^8^ for 5 intratumoral inj. in one patient.	Headache, gait disturbance, tongue deviation, fatigue	Median overall survival of 10.3 months
NCT02208362; 2015- ? Active, not recruiting	IL-13Ra2 (20)	1	Second-generation (4-1BB) CAR on memory T cells	(2–10) × 10^6^ for 16 inj. (6 intra-cavitary inj., 10 intra-ventricular inj.).	Not reported	Complete remission for 7.5 months
NCT02209376; 2014-2018; Terminated*	EGFR-vIII (22)	10	Second-generation (4-1BB) CAR on CD8+ T cells	(1.75–5) × 10^8^ for single intravenous inj.	Nervous system disorders (facial muscle weakness, epilepsy, headache, brain edema, and intracranial hemorrhage), left ventricular systolic dysfunction, skeletal muscle weakness	Median overall survival of 8.4 months; one case stayed in stable disease for at least 8 months
NCT01454596; 2012-2019; Completed;	EGFR-vIII (26)	18	Third-generation (4-1BB+CD28) CAR on T cells	6.3×10^6^–2.6×10^10^ for single intravenous inj.	Two cases developed fatal dyspnea (one died of it) after receiving a high-dose CAR-T cell injection (≥3×10^10^ in terms of CD3+ T-cell number); Others: thrombosis, bacteremia, temporary motor dysfunction and urinary incontinence, etc.	Median progression-free survival of 1.3 months, median overall survival of 6.9 months; one case achieved a progression-free survival of 12.5 months and overall survival of more than 59 months
NCT01109095; 2010-2018; Completed;	HER2 (21)	17	Second-generation (CD28) CAR on virus-specific T cells	10^6^–10^8^/m2 inj. for 1–6 intravenous inj.	Fatigue, headache, cerebral edema, hydrocephalus, neutropenia, lymphopenia, etc.	Median overall survival of 11.1 months; one case achieved a partial remission for 9 months, seven cases stayed in stable disease for 2–29 months

^*^Sponsor decision to terminate prior to completion to pursue combination therapies.

Despite the promising results *via* systemic administration, more and more researchers recently prefer regional CAR-T cell therapies in treating solid tumors (23). Especially in anti-glioma CAR-T cell therapies, 14 out of 24 NCT (National Clinical Trial)-registered ongoing trials, with known administration routes, exploit intraventricular and/or intracavitary delivery as administration routes (**Supplementary Table 1**). Unlike hematologic malignancies and other solid tumors, brain tumors present a unique challenge for T-cell infiltration due to the presence of BBB. Although early trials have demonstrated successful trafficking of T cells into tumor, due to the partial disruption of BBB in brain malignancies (21, 22), mounting evidence has shown that locoregional delivery of CAR-T cells, e.g., intratumor/intracavitary and intraventricular administration, can bypass BBB, allow direct access to the tumor site, and thus present more potent antitumor efficacy and less systemic toxicities as compared with systemic administration (24, 25). Noteworthily, systemic administration can induce sequestration of the infused T cells in lung, which not only limits intratumor T-cell infiltration/activation (23), but also may lead to fatal adverse events (26).

Regarding safety, all the published trials (**Table 1**) have shown that CAR-T cell therapies in the routine dose, either through locoregional delivery or through systemic intravenous infusion, are rather safe in treating gliomas, as nearly all adverse events are less than CTCAE grade 3, and most are headache, fatigue, and self-limiting nervous system signs (epilepsy, gait disturbance, etc.) (2, 20–22, 26). The cytokine release syndrome, which is commonly seen in CAR-T cell therapies on hematological malignancies, is quite rare in the treatment of GBMs (2, 20–22, 26). However, extremely high-dose CAR-T cell administration (≥3×10^10^ CAR-T cells) through systemic infusion is reportedly related to a fatal syndrome, which appeared as severe acute dyspnea, hypoxemia, and hypotension, in two patients with recurrent GBMs (one patient died of it within 4 h after the onset) after they received the EGFR-vIII targeted CAR-T cell therapies (26). Considering the target, EGFR-vIII is a tumor-specific antigen that is absent in normal tissues; this syndrome is unlikely attributed to the off-target toxicity. The pulmonary venostasis induced by the high-dose infusion of T cells would be an arguable cause for the syndrome, since the autopsy on the patient shows significant pulmonary edema.

Although CAR-T cell therapies on GBMs have present encouraging outcomes preclinically and in some early-phase trials, the successful bench-to-bedside translation of this novel therapeutic still faces several challenges, including the short duration of clinical remission (2, 20–22, 26), quick clearance of infused CAR-T cells in blood (21, 26), limited proportions (<5%) of systemically infused CAR-T cells that migrated into the brain (22), antigen loss due to tumor heterogeneity (2, 20, 22), as well as extensive immunosuppressive microenvironment within GBMs (22). Therefore, a variety of innovative strategies in CAR designs as well as clinical trial designs have been attempted to overcome these challenges (27, 28). Among these strategies, we believe that targeting a surface antigen or a group of surface antigens that are expressed in the majority of glioma cells, engineering CAR-T cells to overcome the severe immunosuppressive tumor microenvironment (TME), and editing CAR-T cells to sustain its antitumor functions *in vivo* are three major aspects that needed to be considered to develop the next generation of CAR-T cell therapies against this deadly brain cancer.

## Tumor Heterogeneity and Target Selection

GBM is notorious for its high intratumor heterogeneity, which is revealed by studies of sequencing multi-site samples from one tumor as well as single-cell sequencing (29–32). Several theories, including clonal evolution (33), cancer stem cell model (34), and interclonal cooperativity (35), have attempted to explain the origin, formation, and dynamics of intratumor heterogeneity from different perspectives (36). Intratumor heterogeneity not only leads to chemotherapeutic or targeted therapeutic resistance, but also contributes to short-term recurrence, and thereby treatment failure after CAR-T cell therapies. Several preclinical studies have identified antigen loss, which is the expression of target antigens decreasing while non-target antigen expression increases after immunotherapy, and is a major cause of immune escape in GBM orthotopic murine models receiving CAR-T cell treatment (37, 38). Meanwhile, early trials of IL-13Rα- or EGFR-vIII-targeted CAR-T cell therapies also reveal the downregulation or even absence of target antigens in recurrent tumors after treatment (2, 22). Therefore, overcoming intratumor heterogeneity remains paramount in CAR design to improve therapeutic efficacy.

**Figure 2:**
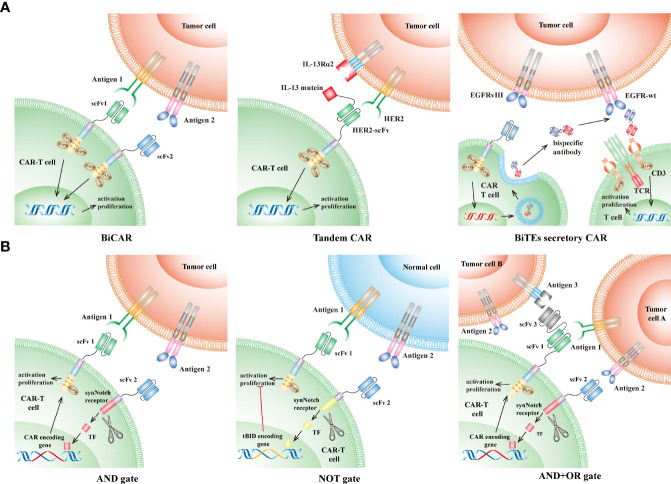
CAR designs to overcome the GBM intratumor heterogeneity. **(A)** CAR targeting multiple antigens: (1) Bispecific CAR (BiCAR, middle): BiCAR-T cells co-express two CARs that target different antigens on tumor cells. (2) Tandem CAR (Tan-CAR, right): Tan-CAR joins two antigen-binding domains to make a tandem CAR exodomain that can be activated by encountering either or both different antigens, e.g., HER2 and IL13Rα2. (3) Bispecific T-cell Engagers (BiTEs) secretory CAR (left): BiTEs are composed of two distinct arms: one arm targeting the wild-type EGFR on tumor cells and another arm specifically binding to the CD3ϵ subunit on endogenous T cells. BiTEs-CAR T cells can directly kill tumor cells that express EGFR-vIII, while indirectly redirecting endogenous T cells to eliminate tumor cells expressing wild-type EGFR through secreting BiTEs. **(B)** Logic-gate principle: (1) “AND” gate (left): the intracellular domain of one CAR is designed as a synNotch receptor structure that is cleaved to form a transcription factor after engaging antigen 1 and subsequently initiate the expression of another CAR specific for antigen 2. Thus, the tumor-killing effect can only be achieved when the CAR-T cells encounter tumors cells simultaneously expressing both antigen 1 and 2. (2) “NOT” gate (middle): similar to the “AND” gate design, except that activation of one CAR (targeting antigen 2) leads to suppressing the activation of another CAR (targeting antigen 1). Therefore, these CAR-T cells can be activated only if they encounter antigen 1 without the presence of antigen 2. (3) “AND+OR” gate (right): the first CAR is designed as in the “AND” gate design, and the following expressed CAR is a TanCAR structure (“OR” gate), which can be activated when it encounters antigen 1 or 3. CAR, chimeric antigen receptor; TCR, T-cell receptor; scFv, single-chain variable fragment; TF, transcription factor; tBID, truncated BH3 interacting death agonist.

To date, a variety of strategies in CAR design have been developed to limit the antigen escape led by intratumor heterogeneity. Although these strategies are quite distinct in detail, most are focused on spanning recognition of CAR to two or more antigens, which would significantly increase tumor-cell-killing coverage, and thus avoid or delay the antigen escape (39–44) (**Figure 2A**). Bispecific CAR (BiCAR) refers to bivalent CAR-T cells co-expressing two CARs that target different antigens on tumor cells. Tandem CAR (Tan-CAR) is another bivalent CAR, in which two antigen-binding domains, joined as a tandem CAR exodomain, share a common intracellular signal transduction domain that can be activated by encountering either or both different antigens. Hegde et al. showed that Tandem CAR-T cells, targeting HER2 and IL13Rα2, can mitigate antigen escape, display enhanced antitumor efficacy, and thus improve survival in a murine GBM model (42). They also observed that Tandem CAR-T cells exhibited more sustained but not more exhaustible anti-glioma activities than the corresponding Bispecific CAR T cells (42). Choi et al. developed a Bispecific T-cell Engager (BiTE) secretory CAR (40) that targets EGFRvIII while locally releasing BiTEs for engaging endogenous T cells against the wild-type EGFR, which is not only frequently overexpressed in GBMs but also expressed in organs such as skin tissues. They observed that BiTE CAR displayed superior activity in eliminating heterogeneous tumors over the monovalent EGFRvIII-CAR while avoiding the on-target and off-tumor toxicity against human skin grafts (45). The Trivalent CAR-T, designed to target three antigens simultaneously (IL-13Rα2, HER2, and EphA2), exhibited more powerful and broader tumor-killing capacity than the BiCAR. Nevertheless, the proportion of IL-13Rα2-HER2-EphA2 three negative cells in a few patients is approximately 20%, which would also lead to antigen loss in these patients (39).

Recently, more than ten CAR target antigens have been identified in GBMs (**Figure 1**) and have shown promising preclinical results (37, 46–57). However, compared with the considerable tumor-specific proteome, it is reasonable that a large number of other antigens, which are possibly fit for CAR-T cell therapies, remain to be discovered. Therefore, large-scale discovery for CAR antigens in GBMs is warranted. Screening potential target antigens by comparing public omics data between tumors and the adjacent brain tissues, followed by validating them through high-throughput protein assays will accelerate the process of discovery. In particular, the strategy of logic gates, which has recently been used in CAR design to target two or more antigens (**Figure 2B**) (58, 59), will significantly increase tumor-cell-killing coverage while minimizing the off-target toxicity for the GBM treatment (60, 61). The “AND” gate refers to CAR-T-cell activation achieved in the presence of both antigens, whereas the “NOT” gate represents the activation suppressed when both antigens are present. Both gates utilize a synNotch receptor structure as a molecular switch to trigger the expression (AND) or inhibit the function (NOT) of the second CAR, which is specific for another antigen (**Figure 2B**). Tandem CAR (Tan-CAR) is actually an “OR” gate design that can be integrated with the “AND” gate strategy (**Figure 2B**). By integrating the strategy of logic gates and machine learning method, a huge number of potential combinations in known CAR targets were optimized to improve recognition selectivity of CAR-T cells against 33 different kinds of tumors (58, 59). Among them, GLRB-CD56 is a target combination in which both targets are overexpressed in GBMs but not expressed in the same normal tissue (59). Therefore, the “AND” gate strategy can be utilized to target this combination for improving CAR recognition specificity, thereby facilitating the development of novel CAR-T cell therapy against GBMs.

Nevertheless, the new potential CAR targets or target combinations identified from these strategies may still encounter the following problems (1): These strategies are usually based on the public gene transcriptional data, which sometimes did not represent the authentic protein expression of targets (2). The spatial distribution of targets within the tumor cannot be reflected by the level of target gene expression (3). Concerning the off-target toxicities, it is rather tricky to select the targets that are overexpressed in tumors but also moderately expressed in normal tissues. These targets are more ubiquitous than those absent in normal tissues. Although some of these targets such as HER2 have already been shown as a safe CAR target in treating GBMs in an early-phase trial (21), novel CAR-T designs are still required to further minimize the possibility of off-target toxicities (62, 63) (4). These strategies omit the structural difference of antigen between tumors and normal tissues, which can also be targeted. One example is a CAR design that targets a cryptic epitope, the 287–302 amino acid loop in EGFR, which is only exposed and recognized by CAR when the protein is activated, mutated, or overexpressed (tumor cells), whereas the recognition is blocked when the protein is in an inactivated or wild-type status (normal tissues) (64, 65). As a result, the CAR exhibits strong *in vivoin-vivo* and *in vitro* tumor-killing capacity against EGFR-vIII mutant or EGFR-overexpressing tumor cells, but maintains low toxicity to EGFR normally expressed cells (43). Therefore, the target information at the protein level pertaining to its protein expression and structure is necessary. Recent advances in protein expression analysis by proteomic technologies (66) as well as innovations in protein structure prediction by artificial intelligence (67, 68) will greatly promote the discovery of novel CAR targets for the GBM treatment.

GBM exhibits strong plasticity in tumor evolution and will adaptively inhibit target gene expression after treatment, which could lead to immune escape, even if the target could exist in all glioma cells (2). In order to overcome this kind of immune escape, there are two approaches that can be utilized (1): Forcibly expressing targets in tumor cells *via* gene therapies: Anthony et al. engineered an oncolytic virus to express a nonsignaling, truncated CD19 protein in solid tumors, which can be selectively targeted by CD19-CAR-T cells (69). Obviously, this approach is greatly limited by the transfection efficiency of virus (2). Enhancing antigen spreading *via* cytokine-released CAR-T cells (the fourth-generation CAR-T cells): CAR-T cell-mediated tumor killing can promote a bystander-killing effect of endogenous T cells against untargeted tumor cells, termed antigen spreading (70, 71). This phenomenon can be further enhanced by the fourth-generation CAR-T cells releasing cytokines such as Flt3L, a DC chemotactic cytokine (72). A preclinical study has demonstrated their improved efficacy against tumor models with heterogeneous antigen expression (72). However, this bystander-killing effect is exerted by endogenous T cells, which are also significantly affected by the highly immunosuppressive microenvironment of GBM, their severe exhaustion states, as well as the distinctive sequestration effect on T cells by brain tumors (73, 74). Therefore, the intensity, persistence, as well as clinical significance of this bystander-killing effect remain poorly understood, thus requiring further investigation in preclinical models and clinical studies (75, 76).

## Highly Immunosuppressive Tumor Microenvironment Within GBMs

**Figure 3:**
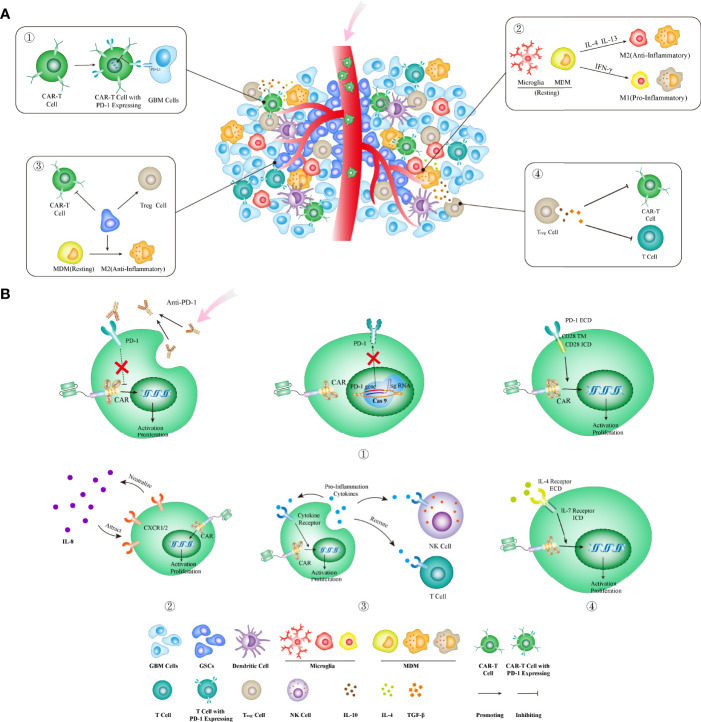
Strategies to overcome the highly immunosuppressive GBM microenvironment. **(A)** The immunosuppressive microenvironment can limit CAR-T cell functions through several ways: ① upregulating PD-1 and other immune checkpoint molecules; ② increasing IL-4 and IL-13 that promote the transformation of TAMs into anti-inflammatory phenotype; ③ existence of GSCs that directly and indirectly (through activating Treg cells and M2-type TAMs) inhibit CAR-T cell functions; ④ increasing the infiltration of Treg cells that directly and indirectly (through secreting IL-10, TGF-β, etc.) suppresses CAR-T cell functions. **(B)** The corresponding strategies in CAR designs to block or reverse these immunoinhibitory effects. ① Three methods for blocking the PD-1 pathway: combining with antibodies blocking the PD-1 molecule (left), knocking out the *PDCD1* gene (encoding PD-1) by the genome editing method (middle), and using a PD-1 chimeric switch receptor (right) that reverses the inhibitory signal by PD-1 activation into the stimulatory signal within CAR-T cells; ② CAR-T cells armed with IL-8 receptor (CXCR1 and CXCR2) could be attracted into tumors enriched with IL-8 and neutralized its immune-inhibitory effect; ③ the fourth-generation CAR-T cells armed to secrete proinflammatory cytokines that can enhance the direct tumor-killing activities by CAR-T cells as well as the indirect bystander killing by endogenous T cells; ④ inverting the inhibitory effects of anti-inflammatory cytokines through transgenic expression of an inverted cytokine receptor that fuses the IL-4 receptor exodomain with the IL-7 receptor endodomain that activates T cells. MDM, myeloid-derived macrophages; ECD, extracellular domain; TM, transmembrane domain; ICD, intracellular domain; GSC, glioma stem cell.

Mounting lines of evidence have supported the view that the highly immunosuppressive TME formed within GBMs can locally and systemically damp the cancer-killing effect exerted by CAR-T cells (**Figure 3A**). The major cellular component of TME within GBMs is tumor-associated macrophages (TAMs), which include microglia (MG) and myeloid-derived macrophages (MDMs). TAM densities have been proven to be associated with worse outcome and increased malignancy in gliomas (77, 78). Several studies have revealed that high-grade gliomas exhibited significantly increased infiltration of MDMs, which highly express the genes related to immune suppression as well as anti-PD1 therapy resistance (79, 80). Treg cells play a crucial role in maintaining the immune inhibition of TME and suppressing CD8^+^ T cell-mediated immune response. Treg cell infiltrative levels increased significantly in GBM after anti-EGFRvIII CAR-T cell treatment (22). Treg cells also secreted cytokines such as IL-10, IL-4, and IL-13, thus promoting the development of TAMs with immunosuppressive properties (76, 81). Glioma stem cells (GSCs) also play an important role in recruiting monocytes and promoting their transformation toward tumor-promoting phenotypes, thus directly or indirectly (*via* Treg cells activation) inhibiting effector T-cell activation/proliferation while inducing their apoptosis (82, 83). Meanwhile, immune checkpoint molecules, such as PD-1, LAG3, TIM-3, and TIGIT, are highly expressed on T cells infiltrating GBMs (84, 85); immunosuppressive molecules such as IDO1, PD-L1, and IL-10 will compensatorily increase after CAR-T cell treatment (22). All these cells and molecules constitute the highly immunosuppressive TME that will locally reshape the infiltrating antitumor T cells and thus limit their activation and proliferation. Interestingly, GBMs can promote T-cell sequestration in bone marrow through downregulating a T-cell receptor—S1P1, which is essential for lymphocyte recirculation (73). Therefore, tailoring CAR-T cells to overcome the impact of immunosuppressive TME is paramount for developing CAR-T cell immunotherapy against GBMs.

Modifying the immunosuppressive molecules on T cells is one strategy commonly used in CAR design to overcome the immunosuppressive effect by the GBM TME. Among these molecules, PD-1 is the most attractive immunosuppressive receptor that has been proven to be a successful immunotherapeutic target in treating cancers. The CAR design, tailored to reduce its immunosuppressive effect, has already exhibited encouraging results preclinically and in early clinical trials (86–89). These designs include combinatory therapy with PD-1 blocking antibodies (clone RMP1-14 and clone UB8-1B9), CAR-T cells that secrete PD-1 blocking antibodies (87, 89), CAR-T cells with the PD-1 gene knockout, as well as a chimeric switch-receptor targeting PD-1 that comprises the truncated extracellular domain of PD-1 and the transmembrane and cytoplasmic signaling domains of CD28 into CAR-T cells (86, 90–92) (**Figure 3B**).

However, considering that PD-1 usually functions as a “braker” for excessive T-cell activation, the safety concerns regarding suppressing the PD-1 pathway during CAR-T treatment should not be ignored. Moreover, Treg cells also express PD-1 and thus systemic PD-1/PD-L1 blockade would lead to enhancement of Treg cell function, thereby significantly suppressing antitumor immune responses and causing a state of hyper-progressive disease in gastric cancer (93–95). A regards Treg cells constituting a majority of infiltrating T cells in GBMs, this effect should be carefully monitored in future clinical studies. On the other hand, several studies have revealed that persistent PD-1 blockade will alter the kinetics of T-cell differentiation. In this situation, T cells will proliferate too rapidly, undergo premature differentiation, and lose the effector memory phenotype, thereby generating a large number of terminally differentiated T cells. This phenomenon is more evident in PD-1 knockout T cells (96, 97). Therefore, the combinatory strategy with PD-1 blockade needs further intensive investigation in preclinical models as well as early trials, concerning the complexity of immune checkpoint pathways. In particular, several issues should be addressed before this strategy enters clinical practice: Will PD-1 blockade exhibit other functions during the CAR-T cell treatment? Is PD-1 inhibition beneficial to the survival of CAR-T cells in the long run? Does PD-1 blockade significantly exaggerate the side effects of CAR-T cell therapy? Is PD-1 blockade alone sufficient to control T-cell exhaustion, considering the “super-cold tumor” nature of GBMs as well as the availability of antibodies targeting other immune checkpoints (98, 99)?

Cytokines can be simply subtyped into pro- and anti-inflammatory cytokines, based on their effect on T-cell functions. The pro-inflammatory cytokines, such as IL-12, IL-18, and IL-15, have been used to arm CAR-T cells (the fourth-generation CAR-T cells) to enhance tumor-killing capacity (11, 13, 14). Meanwhile, these cytokines as well as others such as CCL-19 and CCL-21 can also recruit T cells or NK cells to promote the bystander killing on tumors (100, 101). On the other hand, the receptors of anti-inflammatory cytokines can be exploited to entrap their corresponding immune-inhibitory cytokines. For instance, IL-8 plays a vital role in MDSC recruitment into TME, tumor epithelial–mesenchymal transition, angiogenesis, and metastasis (102–104). The expression of IL-8 in gliomas significantly increases after radiotherapy (105). Therefore, CAR-T cells armed with IL-8 receptors, CXCR1/CXCR2, can neutralize IL8’s immunosuppressive effect and promote T-cell infiltration into TME (**Figure 3B**). The 8R70CAR, a CD70-targeted and IL-8 receptor-modified CAR, has exhibited enhanced abilities in promoting CAR-T cell intratumoral trafficking and persistence, thereby contributing to tumor regression and immunologic memory in multiple murine cancer models (105). Inverting the inhibitory effects of anti-inflammatory cytokines is an alternative approach to protect CAR-T cells from the immunosuppressive TME. IL-4 is a type 2 cytokine that usually contributes to the upregulation of anti-apoptotic molecules in malignant cells and suppression of antitumor immune response (106–108). Transgenic expression of an inverted cytokine receptor that fuses the IL-4 receptor exodomain and IL-7 receptor endodomain in CAR-T cells can improve their proliferation, survival, as well as antitumor activity in an IL-4-rich microenvironment (109, 110) (**Figure 3B**).

## *In Vivo* Persistence of CAR-T Cell Therapy on GBMs

Based on current lines of evidence from early trials, the *in vivo* persistence of CAR-T cells in treating GBMs is mostly less than 2 weeks, regardless of administration routes (20–22, 26). The *in vivo* persistence was significantly impacted by several factors, including dosages, preconditions before infusion, inherent characteristics of T-cell stimulatory signaling, and adaptive changes of gene expression profiling in tumor-infiltrating T cells (111, 112). The last two factors decide T-cell phenotype and activation status, and profoundly affect T-cell *in vivo* persistence.

### Tuning Inherent Signaling

**Figure 4:**
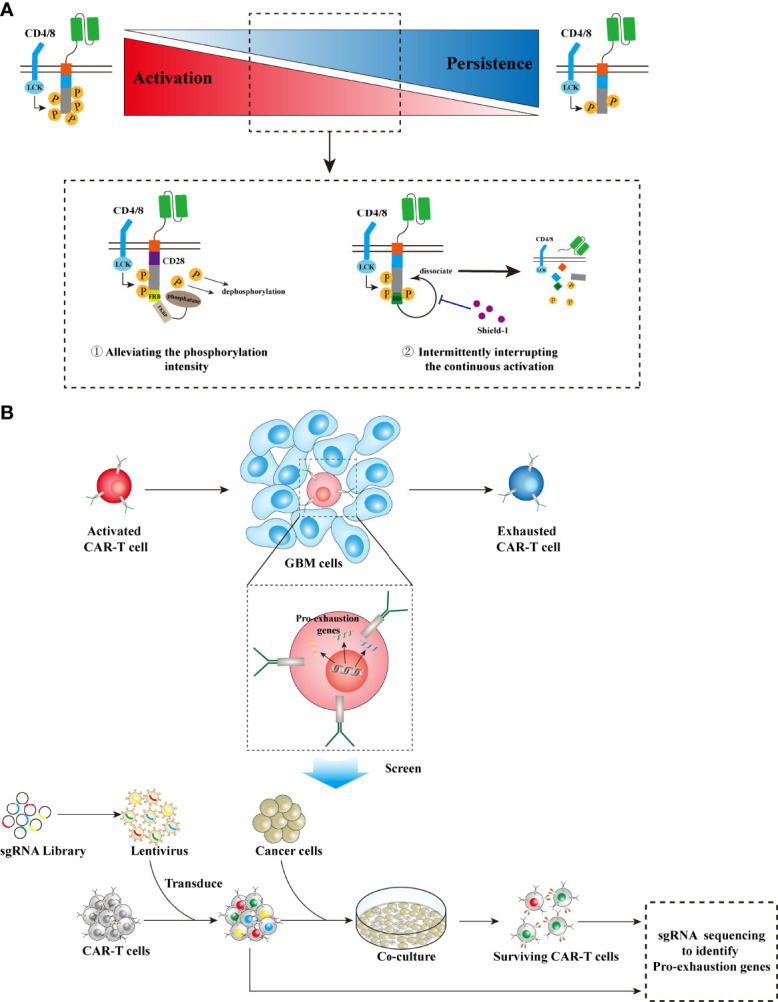
Strategies to prolong CAR-T cell persistence. **(A)** Tuning inherent signaling. High phosphorylation intensity in TCR signaling leads to robust but unsustainable antitumor activity, while alleviating the phosphorylation intensity results in reduced but sustained tumor-killing effects. Two strategies can be utilized in CAR design to tune the phosphorylation intensity, thereby prolonging CAR-T cell persistence while maintaining moderate antitumor activity: ① Integrating CD28-CARs with the FRB domain, which recruits phosphatases *via* binding FKBP and then decreases the phosphorylation level, can suppress CAR overactivation. ② Intermittently administering a small molecular drug, shield-1, to interrupt the dissociation effect on CARs by the DD domain that is fused with CARs, will block CAR continuous activation. **(B)** Screening pro-exhaustion genes: GBM cells and their microenvironment can adaptively alter the gene expression profiles of infiltrative T cells into an exhausted phenotype, leading to shortened persistence. Identification of these pro-exhaustion genes *via* CRISPR-based genome-scale knockout technology will greatly accelerate CAR-T cell development to prolong persistence. LCK, leukocyte-specific protein tyrosine kinase; FKBP, FK506 binding protein; FRB, FKBP-rapamycin binding; DD, destabilizing domain.

CAR-T cell activation is tuned by intracellular phosphorylation cascade. Although enhancing the phosphorylation intensity of CAR intracellular segments may improve the antitumor effect of CAR-T cells (113), this strategy does not always work (114). As mentioned previously, CD28 and 4-1BB exerted a differential effect on CAR-T cell activation and persistence. The *in vivo* persistence of 4-1BB-CAR-T cells was superior to the CD28, which displayed enhanced phosphorylation intensity (7, 115, 116); the third-generation CAR-T cells sometimes exhibited inferior *in vivo* persistence and tumor-killing capacity as compared with the second-generation CAR-T cells (8–10); CAR-T cells with PD-1 silencing tended to differentiate into terminally exhausted T cells (96, 97). Moreover, CAR-T cells engineered with a CD3ζ chain containing three ITAMs exhibited enhanced activation and increased effector phenotypes, as compared with those having a CD3ζ chain comprising only one ITAM. However, the latter showed increased memory phenotypes, enhanced proliferation, as well as prolonged *in vivo* persistence (117). Therefore, it is arguable that moderately reducing the intensity of activation signaling in CAR-T cells would significantly extend their survival, and thus provide an overall benefit in cancer treatment. Alleviating the phosphorylation intensity through recruiting phosphatases will prolong persistence and thus improve therapeutic efficacy for the CD28 second-generation CAR-T cells (118) (**Figure 4A**). Intermittently interrupting the continuous activation of CAR-T cells can reverse T-cell exhaustion, induce memory phenotype, and thus provide overall survival benefits (119) (**Figure 4A**). However, all these strategies require a delicate control of T-cell signaling, and thus their actual performance should be evaluated intensively in preclinical models as well as in early trials, concerning the complexity of intracellular signaling within T cells and complicated interactions between tumor cells and immune cells. In particular, safety needs to be cautiously assessed in real clinical practice, since loss of control in T-cell signaling would contribute to catastrophic cytokine storm on the one hand or lead to therapeutic ineffectiveness and treatment failure on the other hand.

### Countering Adaptive Mechanisms

Tumor cells and their microenvironment can adaptively alter the gene expression profiles of infiltrative T cells into an exhausted phenotype, leading to shortened persistence and T-cell treatment failure. Meanwhile, tumor immunogenicity is another factor that significantly impacts T-cell persistence in antitumor immune response (120). Therefore, an accurate identification and the specific blockade of these altered genes that are vital to T-cell persistence are essential to counter the CAR-T cell treatment failure induced by this adaptive mechanism. With the development of CRISPR technology as well as the establishment of a CRISPR-Cas9 library, scientists can randomly knock out genes at the genome scale and then perturb genes that would control T-cell exhaustion or tumor immunogenicity (121–124) (**Figure 4B**). Utilizing this strategy, Wei et al. identified the gene REGNASE-1 that engages T-cell metabolism as a negative regulator for adoptive T-cell therapy *via* decreasing T-cell persistence (125). Wang et al. uncovered genes, including TLE4 and IKZF2, that are associated with T-cell exhaustion and effector function *via* screening of CAR-T cells, and identified genes, including RELA and NPLOC4, that are essential for tumor susceptibility to tumor killing *via* the reciprocal screening of GSCs (126). Ye et al. performed *in vivo* screening for membrane protein targets in CD8^+^ T cells in mouse models of GBM and identified a few genes, such as Pdia3 and Mgat5, that dampen T-cell effector functions in gliomas (127). Therefore, this cutting-edge CRISPR technology can be used to uncover key genes that engage T-cell persistence. Knocking out these genes will facilitate CAR-T cells that counter extrinsic immunoinhibitory effects on T cells, thereby enhancing their tumor-killing capacity as well as prolonging the persistence in the body, which can ultimately improve the therapeutic efficacy of CAR-T treatment (125–128).

## Conclusion and Outlook

CAR-T cell immunotherapy has greatly changed the landscape of cancer treatment, especially for hematological malignancies, a fraction of which would be cured by this promising therapeutic modality in the foreseeable future. Although CAR-T cell therapy on GBMs is only in its infancy, the advent of cutting-edge biological technologies will accelerate the process to find novel strategies for GBMs. For instance, the method of genome-scale screening *via* CRISPR-Cas9 can significantly shorten the time for discovery of key genes that can be perturbated to enhance CAR-T cell therapeutic efficacy. Meanwhile, a three-dimensional model of glioma organoids (GOs) can be utilized for better preclinical studies on CAR-T cell treatment, since GOs recapitulate the cellular heterogeneity, structure, and functions of primary tissues as compared with primary culture cells. The technology of single-cell sequencing can be applied to accurately reveal the intratumor heterogeneity in glioma cells as well as the other cellular components of TME, and thus provide abundant information for monitoring immune response and predicting therapeutic efficacy during treatment. Therefore, with the advancement of these technologies and the rapid development in novel CAR strategies, we hope that there will be some CAR-T cell therapeutics that will finally be allowed for clinical use to improve the dismal outcomes of GBM patients.

## Author Contributions

PZ and YZ wrote the manuscript. NJ revised the manuscript. All authors contributed to the article and approved the submitted version.

## Funding

The work was supported by The Capital Health Research and Development of Special (2022-2-2047), the Capital Characteristic Clinical Application Project (Z181100001718196), and the National Natural Science Foundation of China (81702451 and 81930048).

## Conflict of Interest

The authors declare that the research was conducted in the absence of any commercial or financial relationships that could be construed as a potential conflict of interest.

## Publisher’s Note

All claims expressed in this article are solely those of the authors and do not necessarily represent those of their affiliated organizations, or those of the publisher, the editors and the reviewers. Any product that may be evaluated in this article, or claim that may be made by its manufacturer, is not guaranteed or endorsed by the publisher.

## Supplementary Material

**Supplementary Table 1** summarizes all the NCT (National Clinical Trial)-registered clinical trials in respect to CAR-T cell therapies on gliomas.
